# A meta-analysis of the effects of probiotics and synbiotics in children with acute diarrhea

**DOI:** 10.1097/MD.0000000000016618

**Published:** 2019-09-13

**Authors:** Bo Yang, Ping Lu, Mei-Xuan Li, Xiao-Ling Cai, Wan-Yuan Xiong, Huai-Jing Hou, Xiao-Qin Ha

**Affiliations:** a Department of Clinical Laboratory, The People's Liberation Arimy Joint Service Support Unit 940 Hospital; b School of Clinical Medicine, Gansu University of Traditional Chinese Medicine (TCM), Lanzhou; c School of Clinical Medicine, Capital Medical University, Beijing; d Evidence Based Medicine Center, School of Basic Medical Sciences, Lanzhou University, Lanzhou, China.

**Keywords:** acute diarrhea, prebiotics, probiotics

## Abstract

**Objective:**

This meta-analysis assessed the effectiveness of probiotics and synbiotics for acute diarrhea (AD) in children and investigated probiotic formulations, types of interventions, and country factors.

**Methods:**

Randomized, double-blind, placebo-controlled trials evaluating the effects of probiotics or synbiotics on AD were analyzed. We followed the recommendations of the Cochrane Handbook and the Preferred Reporting Items for Systematic Review and Meta-Analysis (PRISMA) statement. The risks of systematic errors (bias) and random errors were assessed, and the overall quality of the evidence was evaluated using the Grades of Recommendations Assessment, Development, and Evaluation (GRADE) approach.

**Results:**

The meta-analysis included 34 studies with 4911 patients. Five and 29 studies presented the results of synbiotic and probiotic interventions, respectively. After intervention, the durations of diarrhea (weighted mean difference (WMD) = −16.63 [−20.16; −12.51]) and hospitalization (risk ratio (RR) = 0.59 [0.48; 0.73]) were shorter, the stool frequency on day 3 (WMD = −0.98 [−1.55; −0.40]) was decreased, and the incidence of diarrhea lasting 3 days was lower in the probiotic and synbiotic groups than in the control groups. Furthermore, in the subgroup analyses, synbiotics were more effective than probiotics at reducing the durations of diarrhea and hospitalization, and Saccharomyces and Bifidobacterium were more effective than Lactobacillus at reducing the duration of diarrhea.

**Conclusion:**

This meta-analysis supports the potential beneficial roles of probiotics and synbiotics for AD in children. Further research is needed to determine problems associated with probiotic/synbiotic mixtures and appropriate dosages.

## Abstract 2

Supplemental Digital Content is available in the text

## 1 Introduction

Acute diarrhea (AD) continues to be the second most common cause of morbidity in children worldwide, especially in developing countries.^[1]^ It has been reported that 15% of children under the age of 5 years die as a result of diarrhea.^[2]^ Viral, bacterial and parasitic gut infections are the most common causes of AD and are related to poor sanitation and hygiene and unsafe water supplies^[3]^; other important causes of AD include antibiotics, infections not associated with the gastrointestinal tract, food poisoning, and allergies.^[4]^ Diarrhea of any cause leads to dehydration and serious long-term sequelae, including hemolytic uremic syndrome, Guillain-Barré syndrome, malnutrition and even death if patients are not promptly and correctly treated^[5]^; furthermore, rotavirus is the main cause of diarrhea mortality in children.^[6]^

The relationship between AD and gut microbiota has attracted attention in recent years. The major factors linking gut microbiota to AD have been studied because the gut microbiota plays an essential role in protecting the ordinary function of the intestinal barrier, and disorders of the composition of gut flora have also been observed in patients suffering from AD. David et al.^[7]^ demonstrated that patients with AD have significant alterations in the enteric composition of Bacteroides and Prevotella; however, the definite mechanism by which the intestinal microbiota affect the progression of AD is unclear. Therefore, various other intestinal microbial agents, including probiotics and synbiotics, have been studied in randomized controlled trials (RCTs) to assess their effectiveness. Probiotics are defined as ‘live microorganisms that can confer a health benefit to the host’ by enhancing gut barrier function and restoring the intestinal flora balance,^[8]^ whereas synbiotics are probiotics combined with nondigestible food ingredient fibers that beneficially affect the host's health by selectively stimulating the growth and activity of some genera of microorganisms in the colon. Probiotics have been proposed as a complementary therapy for the treatment of AD;^[9]^ however, Pinto and Petrova^[10]^ recently concluded that adjuvant therapy with probiotics is not beneficial for young children hospitalized with AD. A recent RCT of synbiotics that included 400 individuals indicated that synbiotic supplementation could significantly reduce the duration of diarrhea in children;^[11]^ however, there is no credible evidence regarding whether synbiotics or probiotics have better effects.

A previous systematic review and meta-analysis that included 20 RCTs and 3867 patients reported that the consumption of probiotics reduced the durations of diarrhea, hospitalization and fever in AD patients.^[12]^ However, the included literature did not strictly meet eligibility and exclusion criteria, some of the included trials evaluated patients with persistent diarrhea,^[13,14]^ and the heterogeneity of the results was not analyzed further. In the current meta-analysis, we excluded 6 articles, added 20 articles with 2752 patients and conducted further analyses to assess the efficacy of synbiotics, country (developed and developing countries), probiotic formulation (genus, species, dose, and combination), and type of intervention (probiotics or synbiotics) to explore sources of heterogeneity and to provide sufficient evidence to guide the clinical application of probiotics and synbiotics.

## 2 Materials and methods

We conducted this meta-analysis according to the Cochrane Handbook for systematic reviews of intervention guidelines, the Preferred Reporting Items for Systematic Review and Meta-Analysis (PRISMA) statement (S1), and the Cochrane statistical methods guidelines;^[15,16]^ this article reports the results of a literature search and does not involve any animal, cell or human experimental research. This study did not require ethics approval in China.

### 2.1 Search strategy

Studies were identified by two authors (YB and MXL) in EMBASE, PubMed, Web of Science and the Cochrane Library databases until February 2018 using the terms probiotics, synbiotics, AD, acute gastroenteritis, and children. The details of the search strategy are shown in Appendix 1.

### 2.2 Eligibility and exclusion criteria

Two investigators screened the literature, and the titles and abstracts of each paper were examined. The inclusion criteria were as follows:

clinical trials;

studies related to the effects of probiotics or synbiotics on AD;

studies written in English; and

studies including patients younger than 18 years.

The exclusion criteria were as follows:

(1) studies without relevant outcomes;
(2) studies that were not RCTs;
(3) trials in which synbiotic and probiotic interventions were mixed with other drugs; and
(4) studies for which the data and full text were not available through various methods.

### 2.3 Data extraction

Two investigators (PL and MXL) independently extracted the data from each included article. The following characteristics of the analyzed studies were collected: author, publication year, country, language, diarrhea type, sample size (female/male), age, and type and daily dose of probiotics/synbiotics. The data regarding outcome indicators included the following: durations of diarrhea, vomiting, fever, and hospitalization; stool frequency on day 3; and the incidence of diarrhea lasting 3 days. If the study data were unclear, we contacted the author to obtain clarification.

### 2.4 Risk of bias assessment

Risk of bias was assessed independently by 2 authors (BY and PL), and disagreements were resolved by discussion between the 2 authors when assessing the trials. Risk of bias was based on sequence generation; allocation concealment; blinding of participants, personnel, and outcome assessors; completeness of follow-up; selective outcome reporting; and other biases. Each trial was graded as ‘yes’, ‘no’, or ‘unclear’ with respect to the abovementioned aspects, representing a high risk of bias, a low risk of bias, or uncertain bias, respectively.^[17]^

### 2.5 Statistical analysis

Statistical analyses were performed using R software, version 3.4.2 (Cochrane Collaboration, Oxford, UK) with a random effects model.^[18]^ Continuous outcome variables were assessed with weighted mean differences (WMDs) and 95% confidence intervals (CIs), and dichotomous outcomes were evaluated with aggregated risk ratios (RRs) and 95% CIs. Statistical heterogeneity was evaluated with the χ^2^ test, and the degree of heterogeneity among the studies was measured by the *I*^2^ statistic. An *I*^2^ value greater than 50%^[19]^ indicated the existence of significant heterogeneity.

Subgroup analyses were conducted to evaluate the influence of country, intervention type, probiotic strain, dose, and combination of probiotics on the main overall outcome indicators. Egger test and Begg test were used to assess the presence of potential publication bias, and a *P* value < .05 was considered statistically significant.^[20]^ A sensitivity analysis was performed by excluding studies one by one or by excluding studies involving a group of subjects with the same disease. These sensitivity analyses were used to investigate whether the overall pooled results were extremely influenced by any single trial.

### 2.6 Rating the quality of evidence

We used the Grades of Recommendation, Assessment, Development and Evaluation (GRADE) Working Group approach to interpret the findings (Langendam, 2013) and the GRADE profiler (GRADEPRO) to import the data from Review Manager 5.3 (RevMan 5.3) to create 'summary of findings’ tables. These tables provide outcome-specific information concerning the overall quality of evidence derived from the included studies, the magnitude of the effects of the interventions examined and the sum of the available data on the outcomes that were considered.^[21]^

## 3 Results

### 3.1 Study selection

**Figure 1:**
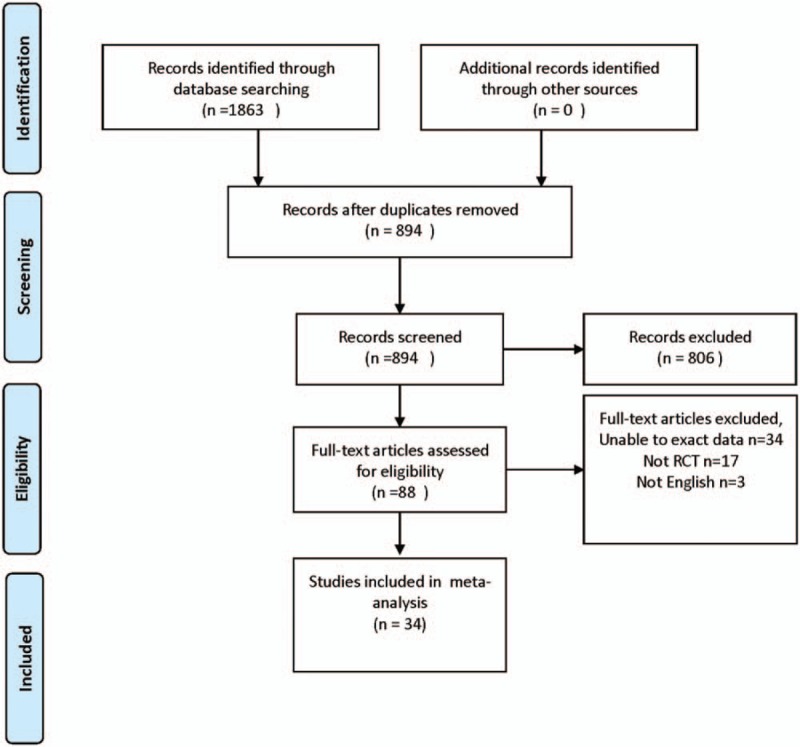
Flowchart of the selection of studies on the effects of probiotics and synbiotics in children with AD.

A total of 1863 studies were included after an initial search, of which 806 studies did not fulfill the inclusion criteria as determined by screening the titles and abstracts. Sixty-six potential trials were selected for full-text assessment, and 32 studies were excluded (3 were not in English, 34 trials had data that could not be extracted, and 17 were not RCTs). Thus, a total of 34 RCTs were included in the meta-analysis. The detailed selection process is presented in Figure 1.

### 3.2 Characteristics of the included studies

The 34^[10,11,22–53]^ included RCTs involved a total of 4911 individuals and were published between 2007 and 2016. Five studies^[10,28,32,34,35]^ presented the results of synbiotic interventions, and probiotic interventions were reported in 29 trials^[11,22–27,29–31,33,36–53]^; 18 studies^[22–27,32,34,36,39,44–47,49–51]^ used a single probiotic strain, and the other 11 studies^[11,28,31,33,35,37,38,40,43,48,53]^ used a mixture of probiotics including 2 to 4 strains. In total, the 6 genera of probiotics used in the studies included Lactobacillus, Bifidobacterium, Saccharomyces, Clostridium, Streptococcus, and Escherichia. *Lactobacillus acidophilus* was used in ten studies. The daily dose of probiotics in these trials ranged from 1.5 × 10^6^ to 1.5 × 10^11^ bacteria (Table 1).

**Table 1:**
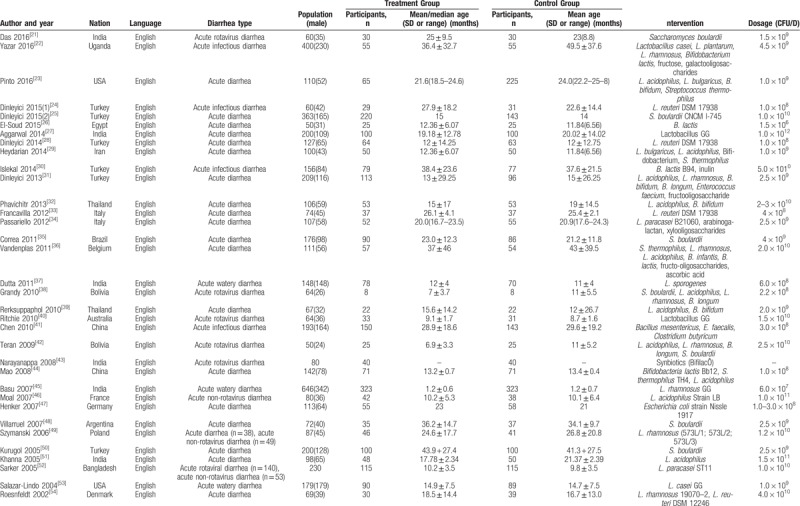
Relevant methodological features and characteristics of the included studies.

### 3.3 Risk of bias assessment

**Figure 2:**
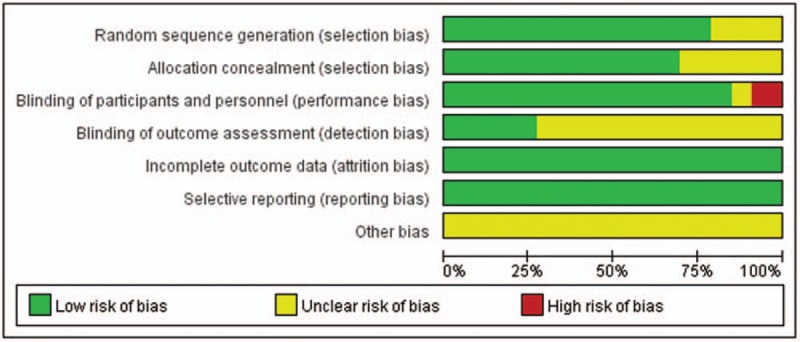
Bias risk assessment of the RCTs on the effects of probiotics and synbiotics in children with AD.

The risk of bias assessment for the 34 RCTs included in this study is shown in Fig. 2. Twenty-seven studies used a proper means of randomizing the participants, and 24 RCTs described how the subjects were concealed for allocation. In 33 trials, patients and caregivers were blinded, but 4 trials did not describe the blinding process, leading to an unclear risk of performance bias. The quality of selective reporting and incomplete outcomes was high in all the RCTs (Fig. 2).

### 3.4 Main outcome indicators

#### 3.4.1 Duration of diarrhea

Twenty-eight studies^[11,22–27,29–31,33,36–45,47–53]^ were included in the pooled analysis of the effects of probiotics and synbiotics on the duration of diarrhea, including 2008 individuals allocated to treatment groups and 1875 individuals allocated to control groups. The pooled results suggest that probiotic and synbiotic supplementation can considerably reduce the duration of diarrhea in children with AD (WMD = −16.63, 95% CI: −20.16 to −12.51; *P* < .001) (Fig. 3 a). There was significant heterogeneity among trials (*I*^2^ = 95%, *P* < .001). The sensitivity analysis suggested that the results of our meta-analysis were stable (Table 2).

**Figure 3:**
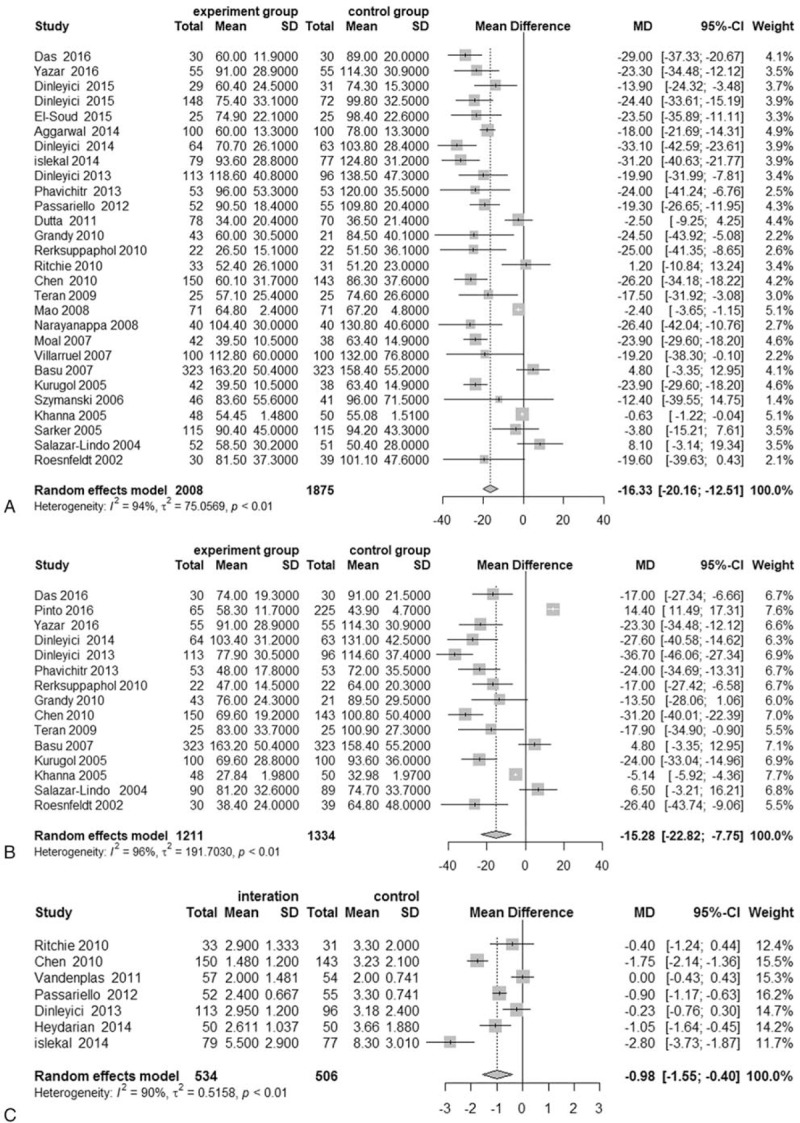
Meta-analysis results for probiotics and synbiotics in AD. Meta-analysis results of (a) the duration of diarrhea in AD, (b) the duration of hospitalization, (c) the duration of stool frequency at 3 days, (d) diarrhea lasting 3 days, (e) the duration of fever, and (f) the duration of vomiting in children with AD.

**Figure 3 (Continued):**
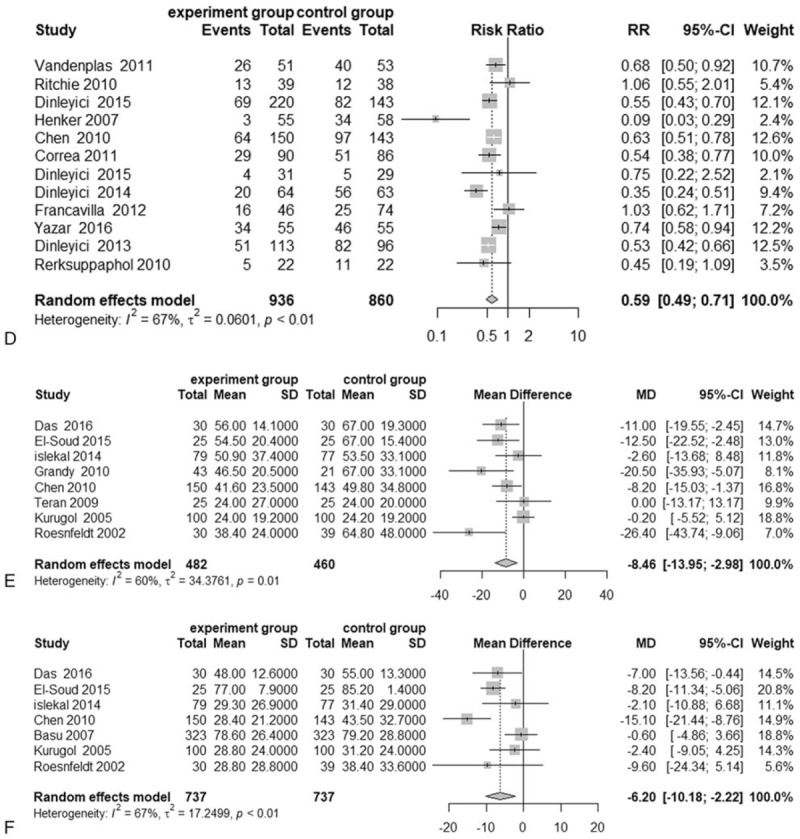
Meta-analysis results for probiotics and synbiotics in AD. Meta-analysis results of (a) the duration of diarrhea in AD, (b) the duration of hospitalization, (c) the duration of stool frequency at 3 days, (d) diarrhea lasting 3 days, (e) the duration of fever, and (f) the duration of vomiting in children with AD.

**Table 2:**
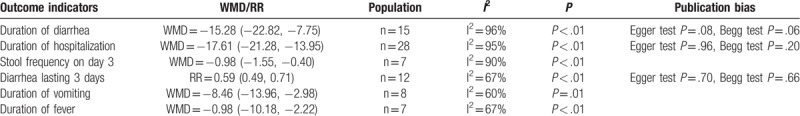
Outcome indicators and publication bias of studies on the effects of probiotics and synbiotics in children with acute diarrhea.

#### 3.4.2 Duration of hospitalization

Sixteen studies^[10,11,22,27,30,31,37,38,40,41,44,49,50,52,53]^ were included in the meta-analysis of the effects of probiotics and synbiotics on the duration of hospitalization, including 1211 patients assigned to treatment groups and 1334 patients assigned to control groups. The aggregated results suggest that probiotic and synbiotic supplementation can significantly reduce the duration of hospitalization in children with AD (WMD = −16.28, 95% CI: −22.82 to −7.75; *P* < .001) (Fig. 3 B), and there was a high degree of heterogeneity (*I*^2^ = 96%, *P* < .001). The sensitivity analysis suggested that the results of our meta-analysis were stable (Table 2).

#### 3.4.3 Stool frequency on day 3

In 7 trials^[28–30,33,35,39,40]^, including an aggregate total of 1040 patients, the researchers reported stool frequency on day 3 after intervention. A pooled analysis of the data from these studies revealed that probiotics and synbiotics could decrease the stool frequency in children with AD on day 3 (WMD = −0.98, 95% CI: −1.55 to −0.40; *P* < .001) (Fig. 3 C), and there was significant heterogeneity among the trials (*I*^2^ = 90%, *P* < .01). The sensitivity analysis suggested that the results of our meta-analysis were stable (Table 2).

#### 3.4.4 Diarrhea lasting 3 days

A total of 11^[11,23,24,27,30,32,34,35,38–40,46]^ studies with 1620 individuals reported the number of children with diarrhea lasting 3 days. When these data were statistically aggregated, there were significantly fewer children with diarrhea in the treatment groups than in the control groups (RR = 0.59, 95% CI: 0.48 to 0.73; *P* < .0001) (Fig. 3 D), and the heterogeneity of these results was high (*I*^2^ = 70%, *P* < .01). The sensitivity analysis suggested that the results of our meta-analysis were stable (Table 2).

### 3.5 Secondary outcome indicators

#### 3.5.1 Duration of vomiting

A total of 7 studies with 1474 individuals reported the duration of vomiting in children with AD. There was a significant decrease in the duration of vomiting in the treatment groups (WMD = −6.20, 95% CI: −10.18 to−2.22; *P* < .001) (Fig. 3 E), and the heterogeneity of this result was high (*I*^2^ = 67%, *P* < .01). The sensitivity analysis suggested that the results of our meta-analysis were stable (Table 2).

#### 3.5.2 Duration of fever

A total of 8 studies with 942 patients reported the duration of fever in children with AD. The pooled results from these studies suggested that probiotics and synbiotics could reduce the duration of fever (WMD = −8.46, 95% CI: −13.95 to −2.98; *P* < .001) (Fig. 3 F), and the heterogeneity of this result was slightly high (*I*^2^ = 62%, *P* = .01). The sensitivity analysis suggested that the results of our meta-analysis were stable (Table 2).

### 3.6 Subgroup analyses

In the subgroup analyses, there were differences among subgroups based on dosage and probiotic combinations. Synbiotics were more effective than probiotics at reducing the durations of diarrhea and hospitalization.

*Saccharomyces boulardii* and Bifidobacterium were more effective than Lactobacillus at reducing the duration of diarrhea. Lactobacillus had no effect on the duration of hospitalization or the incidence of diarrhea lasting 3 days; however, *Saccharomyces boulardii* alone had significant beneficial effects on those outcomes. Results from different countries revealed that the children in developing countries had beneficial effects in terms of the main outcome indicators. However, among the children in developed countries, there were no significant effects of probiotic or synbiotic use (Table 3).

**Table 3:**
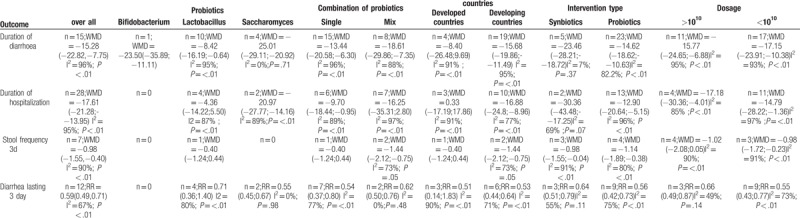
Subgroup analysis and of on the effect of probiotics and synbiotics in children with acute diarrhea.

### 3.7 Publication bias

Egger test and Begg test were used to quantitatively assess publication bias. No publication bias was found in the outcomes, including duration of hospitalization (Egger test *P* = .96, Begg test *P* = .20), duration of diarrhea (Egger test *P* = .08, Begg test *P* = .06) and diarrhea lasting 3 days (Egger test *P* = .70, Begg test *P* = .66). These findings indicate that no obvious publication bias influenced the pooled outcomes (Table 2).

## 4 Discussion

### 4.1 Findings and interpretations

In this meta-analysis, the efficacy of probiotics and synbiotics for the treatment of acute rotavirus diarrhea in children was investigated by reviewing several studies, which yielded contradictory results. The results of the present study showed that probiotics had positive effects on reducing the durations of diarrhea and hospitalization in children compared with control treatments; the duration of vomiting was also reduced in the treatment groups, which was a new finding. Furthermore, significant beneficial effects of treatment with probiotics and synbiotics on stool frequency and the incidence of diarrhea lasting 3 days were indicated in our meta-analysis. Moreover, 2 studies indicated that more beneficial effects were found in rotavirus-positive patients treated with probiotic supplements than in those with other causes of diarrhea,^[34,48]^ and the beneficial efficacy of probiotics and synbiotics was not affected by storage temperature.^[38]^

Previous studies^[54,55]^ have revealed that the mechanisms of action of probiotics and synbiotics likely involve interactions with the intestinal flora, such as by regulation of intestinal immunity, the creation of microbiota that inhibit the amplification of enteric pathogens, or direct strengthening of epithelial barrier function. In a recent study, Zhou et al^[56]^ reported a considerable increase in the diversity of the intestinal microbiota and inhibition of *E coli* probiotic groups in rabbits with diarrhea. Furthermore, TNF-α was upregulated, IL-4 was downregulated, and the intestinal barrier was enhanced in the intestinal tissues of the treatment group. Li et al^[57]^ also reported the successful treatment of diarrhea with fecal microbiota transplantation (FMT).

In the subgroup analyses, we revealed that synbiotic treatment was more effective than probiotic treatment for reducing the durations of diarrhea and hospitalization. Synbiotics beneficially affect host health by producing prebiotics that can improve the survival rate of probiotic compounds during passage through the upper intestinal tract. However, there were no significant differences between probiotics and synbiotics in terms of stool frequency or the incidence of diarrhea lasting 3 days. Yaza et al^[11]^ found no significant differences between a synbiotic group and a group treated with zinc, but the relatively low number of investigations involving synbiotics may have influenced these results, and more RCTs are needed to confirm the benefits of synbiotics.

In the current meta-analysis, the subgroup analysis revealed that different doses are effective in the treatment of AD. However, no dose effects were found in these outcomes between the low (<10^10^ colon forming units (CFU)/day) and high (>10^10^ CFU/day) doses. The reason may be related to the small gap between the doses and the specific strains used in different probiotics. The same dose of different probiotics may contribute greatly to the different clinical outcomes. A comparison of the effects of 3 doses of Lactobacillus indicated a trend in which high doses may be more effective than low doses in reducing the duration of diarrhea; these findings are similar to those in other illnesses, in which high doses of probiotics yielded more beneficial effects than low doses in improving the renal function of patients with chronic kidney disease (CKD).^[58]^

Different effects may be revealed when the same probiotic strains are used alone or in combination. In our results, no significant differences between combination or single-strain probiotics were observed. The effect of combination versus single-strain probiotics is inconclusive. Chapman et al^[59]^ showed greater efficacy with multistrain probiotics; however, in terms of AD, multistrain probiotics were not better than all single-strain probiotics, and Grandy et al^[37]^ revealed no differences in the duration of diarrhea. Notably, negative effects related to competition among different probiotic strains may occur, and more trials are needed to evaluate how to combine probiotics so that they can act synergistically. In terms of single probiotic strains, we found that Saccharomyces and Bifidobacterium were more effective than Lactobacillus at reducing the duration of diarrhea, and Lactobacillus had no effects on the duration of hospitalization or diarrhea lasting 3 days. The effects of Bifidobacterium did not draw enough attention in the treatment of AD; thus, Lactobacillus has been frequently used in studies of AD.

In addition, we found a trend that 25 of the 34 studies in children were from developing countries, especially India.^[22,26,36,42,44,50]^ The costs of probiotic and synbiotic treatments are low;^[1,31,60]^ therefore, probiotics and synbiotics may be a feasible option for patients in developing countries who desire an efficient and low-cost treatment.

### 4.2 Comparison with other studies

A previous systematic review and meta-analysis that included 20 RCTs and 3867 patients reported that the consumption of probiotics reduced the durations of diarrhea, hospitalization and fever in AD patients.^[12]^ However, eligibility and exclusion criteria were not strictly followed when including different studies, some of the included trials involved persistent diarrhea,^[13,14]^ and the heterogeneity of the results was not analyzed further. In the current meta-analysis, we included 34 RCTs with 4911 patients and conducted subgroups analyses to explore the sources of heterogeneity in the results. We also explored clinically meaningful results, such as the beneficial effects of some single strains of probiotics and the dose effects of different probiotics.

### 4.3 Strengths and limitations

The strengths of our current meta-analysis include the large number of RCTs that were included to analyze the effects of probiotics and synbiotics for the treatment of AD and some remarkable results that were first reported in this study. This meta-analysis is the first to investigate synbiotics for the treatment of AD. We also performed a detailed subgroup analysis to explore some problems that have been attracting attention in the application of probiotics and synbiotics. Finally, we used the GRADE approach to assess the quality of evidence (Fig. S2).

There are several limitations of our study. First, due to the lack of available studies, no effect of probiotics or synbiotics on changes in the intestinal flora was found, and the validity of the anti-inflammatory effects of probiotics and synbiotics in patients with AD was not verified. More research is needed to explore the treatment and dose effects of individual probiotic species. Second, significant heterogeneity existed due to the various countries where the studies were performed, the type of intervention, the probiotic doses, and the genera of probiotics used in the included trials. Third, no meaningful results were found with regard to the dose of probiotics or the use of single-strain versus multistrain probiotics, which are important influential factors associated with the beneficial effects of this treatment.

## 5 Conclusions

This meta-analysis supports the potential role of probiotics and synbiotics in the treatment of AD in children. Synbiotics appear to be more effective at reducing the durations of diarrhea and hospitalization. Saccharomyces boulardii and Bifidobacterium were more effective than Lactobacillus at reducing the duration of diarrhea. However, other problems remain to be solved, such as the use of probiotic/probiotic mixtures and the determination of appropriate doses. More RCTs are needed to determine the potential mechanism of action of probiotics in AD to design a rational treatment strategy before clinical application. Moreover, considering the harmful effects of some probiotics,^[61,62]^ further clinical trials should report adverse effects during treatment.

## Acknowledgments

We would like to thank Liang Yao for his helpful advice and assistance.

## Author contributions

**Conceptualization:** Huai-Jing Hou, Xiao-Qin Ha.

**Data curation:** Bo Yang, Xiao-Ling Cai, Wan-Yuan Xiong.

**Funding acquisition:** Xiao-Qin Ha.

**Investigation:** Xiao-Ling Cai.

**Methodology:** Bo Yang, Mei-Xuan Li.

**Project administration:** Wan-Yuan Xiong, Huai-Jing Hou.

**Software:** Bo Yang, Wan-Yuan Xiong.

**Supervision:** Xiao-Ling Cai, Xiao-Qin Ha.

**Validation:** Wan-Yuan Xiong.

**Writing – original draft:** Bo Yang, Ping Lu, Mei-Xuan Li.

**Writing – review & editing:** Bo Yang, Ping Lu, Mei-Xuan Li.

## Supplementary Material

Supplemental Digital Content

## Supplementary Material

Supplemental Digital Content
