# Supplementary material for: A meta-analysis of the effects of probiotics and synbiotics in children with acute diarrhea
Source: Medicine (Baltimore). 2019 Sep 13;98(37):e16618. doi: 10.1097/MD.0000000000016618 (PMC6750275; doi:10.1097/MD.0000000000016618)
Supplement: Supplemental Digital Content [file medi-98-e16618-s001.docx]

Appendix：

#1：diarrhea

#2：diarrhoea

#3：DIARRHEA, INFANTILE

#4：gastroenteritis

#5： #1 or #2 or #3 or #4

#6: Probiotics

#7: Synbiotics

#8： probiotic*

#9：. synbiotic*

#10：Lactobacillus

#11：lactobacill*

#12：Bifidobacterium

#13; (bifidus or bifidobacter*)

#14: treptococcus thermophilus

#15: streptococcus thermophilus.mp.

#16: streptococc*

#17: Lactococcus

#18: lactococc*

#19: Bacillus subtilis

#20: bacillus subtilis

#21: Enterococcus

#22:enterococcus faec*.

#23: Saccharomyces

#24: saccharomyc*

#25:leuconostoc.

#26: pediococc*

#27:bulgarian bacillus.

#28:(beneficial adj3 bacter*).

#29:dairy.

#30:yog?urt.

#31:kefir.

#32: clostridium.

#33:. or/6-33

#34: random$.

#35:factorial$.

#36:(crossover$ or cross over$ or cross-over$).

#37: placebo$.

#38: single blind.

#39:double blind.

#40: triple blind.

#41:(singl$ adj blind$).

#42:(double$ adj blind$).

#43:(tripl$ adj blind$).

#44: assign$.

#45:allocat$.

#46:crossover procedure

#47: double blind procedure

#48: single blind procedure

#49:triple blind procedure

#50. randomized controlled trial

#51:or/34-50
